# The clinical efficacy and experience of bipedicular percutaneous vertebroplasty combined with postural reduction in the treatment of Kümmell’s disease

**DOI:** 10.1186/s12891-020-3113-z

**Published:** 2020-02-07

**Authors:** Jiang Jiang, Fu-long Gu, Zhong-wei Li, Yi Zhou

**Affiliations:** Department of Orthopedics and Traumatology, Nantong Hospital of Chinese Medicine, Room 502 of Building 1 of Sujian Garden City, Chongchuan District, Nantong, China

**Keywords:** Vertebroplasty, Kümmell’s disease, Osteoporotic vertebral fractures, Bipedicular

## Abstract

**Background:**

Kümmell’s disease is a special type of osteoporotic vertebral fracture that causes chronic low back pain and deformity, which seriously affects the living quality of patients. PVP is commonly used to treat osteoporotic vertebral fractures and can quickly relieve low back pain. So, the objective of this study was to analyze the clinical efficacy and experience of bipedicular percutaneous vertebroplasty combined with postural reduction for the treatment of Kümmell’s disease.

**Methods:**

A retrospective analysis of patients with Kümmell’s disease who underwent bipedicular percutaneous vertebroplasty was conducted from February 2016 to May 2018. Operative time, VAS, bone cement injection volume, cement leakage rate, compression improvement of vertebral front edge and vertebral center, and correction degree of kyphosis were collected and analyzed meticulously.

**Results:**

The operative time was 45.33 ± 7.64 min. The volume of bone cement injected was 5.38 ± 1.33 ml. The compression improvement of vertebral front edge was 7.31 ± 1.21%. The compression improvement of vertebral center was 10.34 ± 1.15% and the correction degree of kyphosis was − 2.73 ± 0.31゜. Bone cement leakage occurred in 6 of 39 patients (15.38%), but no clinical symptoms were observed. The VAS scores were significantly lower at 1 day after the surgery, 6 months and at the last follow-up than before the surgery (*P* = 0.000, respectively). The VAS score was lower at the last follow-up than at 1 day after the surgery (*P* = 0.001).

**Conclusion:**

Bipedicular percutaneous vertebroplasty combined with postural reduction could achieve satisfactory analgesic effect in the treatment of Kümmell’s disease, and restore the height of the vertebral body and improve kyphosis to some extent.

## Background

Herrnann Kümmell found in 1891 that patients had no symptoms for weeks to months after minor spinal trauma, but gradually developed into symptomatic, progressive kyphosis [1]. Chronic low back pain was not alleviated and progressively increased. Furthermore, the severely collapsed vertebral body also causes kyphosis that seriously affect the stability of the spine, accelerate the spinal degeneration, and affect the function of cardiopulmonary and abdominal viscera, which seriously affects the living quality of the elderly [2]. So the literature then used Kümmell’s disease to name the disease. Kümmell’s disease is a special type of osteoporotic vertebral cavity-like fracture that mainly occurs in the thoracolumbar region [3], which is a delayed vertebral collapse caused by osteonecrosis and non-union [4].

Percutaneous vertebroplasty (PVP) has been widely used in the treatment of osteoporotic vertebral compression fractures due to its advantages of the rapid pain relief, the stability of fractured vertebral body, and the partial recovery of vertebral body height [5]. The main analgesic mechanisms of PVP are the recovery of mechanical strength, the reconstructed stability of the fractured vertebrae, and the necrosis of nerve endings in the vertebral body and surrounding tissues caused by the cementation and the chemical toxicity of the bone cement monomer [6]. At present, PVP often use transpedicular approach [7]. But, Unilateral pedicle approach PVP injection with less bone cement and uneven distribution in vertebral body was questioned about its clinical effect and safety [8].

The most literatures reported that Kümmell’s disease is associated with ischemic osteonecrosis and pseudoarthrosis formation [1, 9, 10]. There are obvious fissures and cavities in Kümmell’s disease [11]. The fractured vertebral body can perform telescopic activities in the fractured site. Therefore, there is no need to use the balloon for further expanding the fractured vertebral body, and the postural reduction can effectively restore the degree of vertebral compression and kyphosis [12].

Therefore, we conducted this study to analyze the clinical efficacy and experience of bipedicular PVP combined with postural reduction for the treatment of Kümmell’s disease. The clinical efficacy and experience were meticulously collected and evaluated. The compression improvement of vertebral front edge and vertebral center and the correction degree of kyphosis were observed to assess the restoration of vertebral height and the improvement of kyphosis. The VAS were observed to evaluate pain relief. Bone cement leakage and its clinical symptoms will be observed to assess its safety.

## Methods

**Fig. 1:**
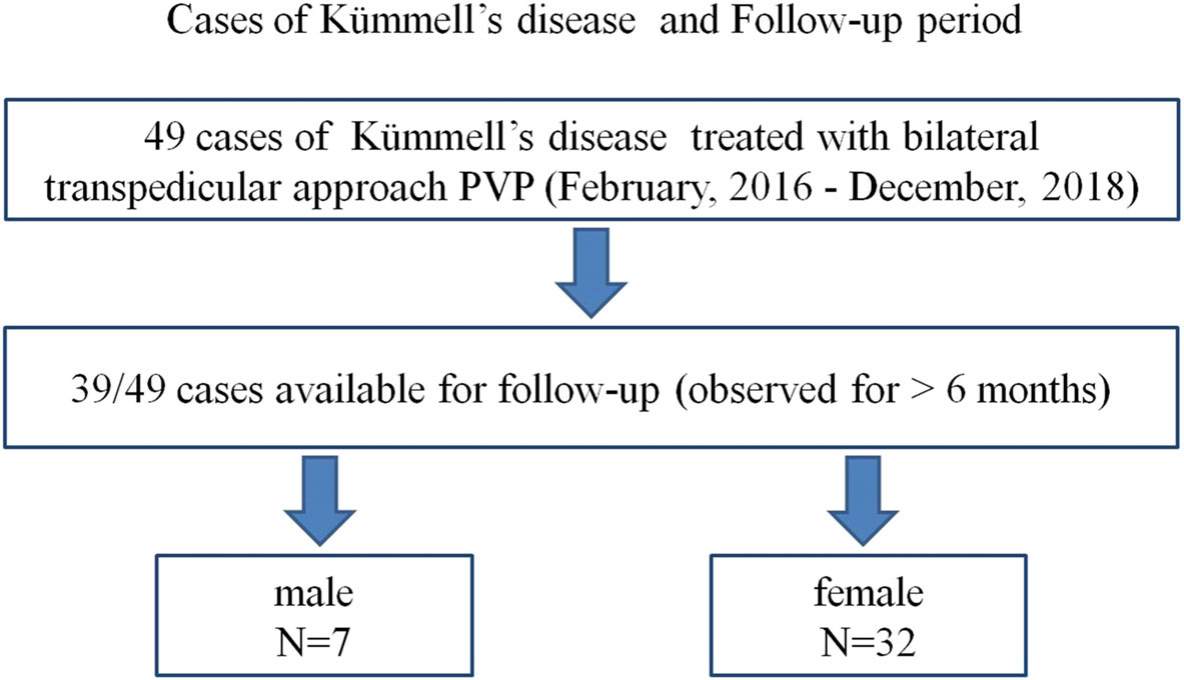
Patients with Kümmell’s disease were treated with bilateral transpedicular approach PVP and followed up more than > 6 months

Patients with Kümmell’s disease who underwent PVP combined with postural reduction in our hospital from February 2016 to December 2018 were enrolled in this study. A total of 49 patients were evaluated (Fig. 1).

The inclusion criteria were as follows: (1) chronic chest and back pain symptoms; (2) age ≥ 55 years; (3) imaging examination showed compression and transverse light transmission areas in the vertebral body or characteristic intravertebral vacuum phenomenon, but the back wall of the vertebral body was intact; (4) the follow-up time was more than 6 months after surgery. The exclusion criteria were as follows: (1) patients with neurological symptoms; (2) patients with malignant tumors or metabolic bone diseases; (3) chronic thoracolumbar pain due to spinal degenerative diseases; (4) patients with other vertebral body fresh fractures or reoccured vertebral body fresh fractures during follow-up.

In the end, 10 patients were excluded, and 39 patients were included in the follow-up. Among them, there were 7 males and 32 females with the average age 75.59 ± 9.67 years old (ranged, 55–94). The sites of the “responsible vertebrae” were in the following thoracic (T) or lumbar (L) vertebrae: T7 (3 cases), T9 (1 case), T11 (5 cases), T12 (13 cases), L1 (10 cases), L2 (1 case), L3 (5 cases), L4 (1 case). Sixteen cases had no obvious trauma, and 23 cases complained of slight sprain or fall history. Low back pain lasted range between 1 and 24 months. All patients had intractable symptoms of low back pain with local tenderness and percussion pain. The pain was obvious when the posture changed, and the pain after resting was relieved. X-ray examination showed that the vertebral body was wedge-shaped or bi-concave. Magnetic resonance imaging (MRI) examination confirmed the “responsible vertebra”. The position with local tenderness and percussion pain was consistent with the “responsible vertebrae”. The study was approved by the Medical Ethics Committee of authors’ Hospital, and informed consent were acquired from all the patients.

**Fig. 2:**
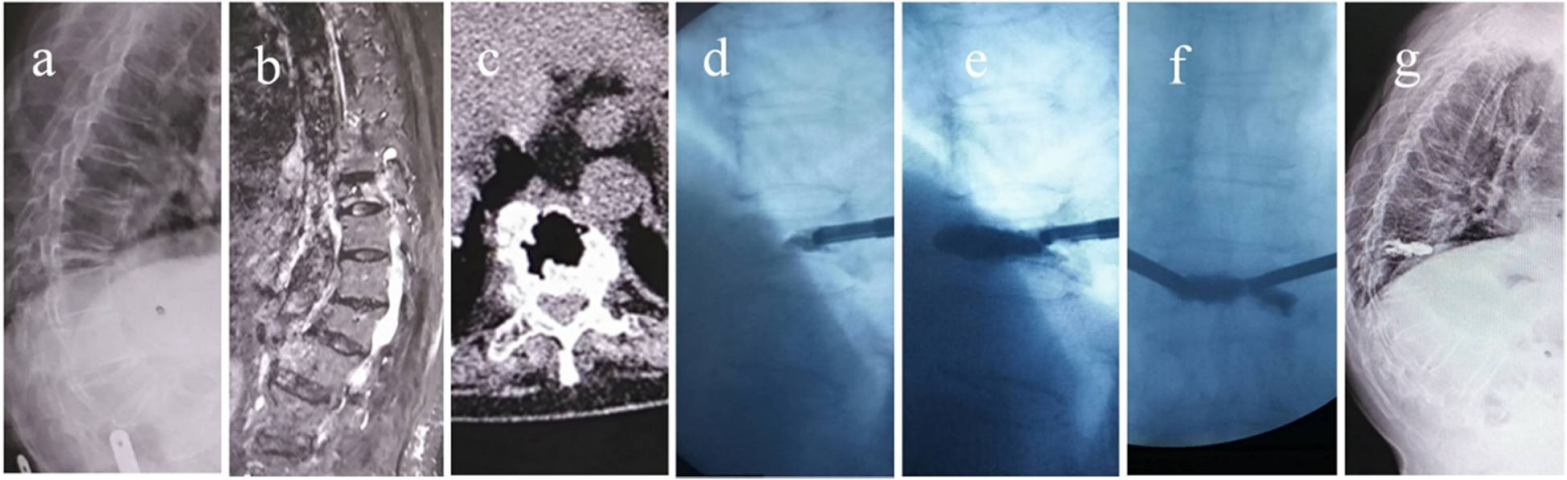
Preoperative, intraoperative and postoperative imaging findings of Kümmell’s disease. **a** Sagittal X-ray image showing the extreme compression and thinning of the diseased vertebrae. **b** Sagittal STIR image showing a band-like low intensity signal. **c** Coronal computed tomography image showing Th8 intravertebral fluid and air-densities. **d** The bone cement injection in the beginning showed that the bone cement penetrates forward. **e** Sagittal C-arm image showed the favorable penetration of bone cement. **f** Coronal C-arm image showed the favorable penetration of bone cement. **g** Sagittal X-ray image postoperatively showed the favorable penetration of bone cement

First, the manual reposition combined with postural reduction was performed to extend the vertebral body for the height restoration. The patients were took the prone position for the suspended chest and abdomen. One assistant used both hands to held the patient’s bilateral armpit from the back side and another assistant held the bilateral feet. Then the two assistants pulled the patient upwards. Simultaneously, the fracture site was pressed down at the injured vertebrae by operator for the maximal lordosis. A side opening guide needle that can adjust the direction of bone cement injection was used for the injection of polymethyl methacrylate cement through the bilateral transpedicular approach (Fig. 2). The penetration orientation of the cement was carefully observed so as to adjust the depth of the needle and the injected direction of bone cement. When C-arm fluoroscopy showed that the bone cement fully blocked the anterior edge of the vertebral body or the upper and lower endplates of the rupture, the bone cement injection should be stopped. On the next day after surgery, the patients worn a thoracolumbar support to get out of bed and was treated with calcium and anti-osteoporosis drugs.

The observed outcomes included the patient’s gender, age, operative time, VAS score, the injection volume of bone cement, the rate of cement leakage, compression improvement of vertebral front edge and vertebral center, and correction degree of kyphosis. VAS scores were performed before surgery, 1 day after surgery, 6 months after surgery, and at the last follow-up. The measurement of kyphosis angle: the lateral x-ray film was streaked on the upper and lower edges of the fractured vertebral body, and the angle between the two lines was the kyphosis angle. The difference between the postoperative and preoperative was the correction degree of kyphosis. Measurement of the degree of vertebral compression: the average value of the posterior margin of the upper and lower adjacent vertebral bodies of the fractured vertebrae on the lateral radiographs was used as the estimated value of the posterior margin height of the fractured vertebral body; then the front edge and central height of the fractured vertebral body were measured; the ratio of the front edge or central height to the estimated height of the posterior margin was used as an evaluation index of compression degree. The front edge and central compression degree of the fracture vertebral body preoperatively and postoperatively were calculated. The differences respectively between the postoperative and preoperative were the compression improvement of vertebral front edge and vertebral center.

SPSS version 19.0 statistical software (IBM Corp., Armonk, NY, USA) was used in statistical analysis. The data were expressed as Mean ± SD. The Levene test was used to test the homogeneity of variance. The VAS scores at different time points were analyzed by Dunnett T3 test. *P* < 0.05 was considered to be statistically significant.

## Results

All patients successfully completed the operation. There were no bone cement adverse reactions and cardiac and cerebrovascular events during and after the operation. No puncture needles entered the spinal canal and injured nerves. The operative time was 35–65 min with an average of 45.33 ± 7.64 min. The injected amount of bone cement was 2.7–8 ml with an average of 5.38 ± 1.33 ml. The compression improvement of vertebral front edge was 3.49–8.91% with an average of 7.31 ± 1.21%. The compression improvement of vertebral center was 7.21–11.98% with an average of 10.34 ± 1.15%. The correction degree of kyphosis was − 2.12--3.64゜with an average of − 2.73 ± 0.31゜.

Cement leakage occurred in 6 of 39 patients (15.38%), but no clinical symptoms were observed. Paravertebral soft tissue and anterior vertebral leakage were reported 2 cases, and intervertebral space leakage was reported 4 cases. No intraspinal and venous leakage was reported.

Patients with low back pain were significantly improved postoperatively compared with preoperatively. After the patient’s pain was relieved, the patient took the lumbar support to get out of bed on the first day after surgery. The VAS scores were significantly lower at 1 day after the surgery, 6 months, and at the last follow-up than before the surgery (*P* = 0.000, respectively). Furthermore, the VAS score was lower at the last follow-up than at 1 day after the surgery (*P* = 0.001). The VAS score decreased at 6 months after the surgery compared with 1 day after the surgery, but its difference was not statistically significant (*P* = 0.050) (Table 1).

**Table 1:** Comparison of VAS score between different time points during the follow-up (Mean + SD)

Time points	VAS scores
Preoperatively At 1 day after the operation Six months after the operation At the last follow-up	6.9218 ± 1.03899 1.6405 ± 0.53118^a^ 1.3679 ± 0.33576^a,c^ 1.2528 ± 0.25158^a,b^

^a^: *P* = 0.000 vs. preoperative VAS score

^b^: *P* = 0.001 vs. VAS score at 1 day after the operation

^c^: *P* = 0.05 vs. VAS score at 1 day after the operation

## Discussion

In our study, the compression improvement of vertebral front edge and vertebral center and the correction degree of kyphosis were obviously improved postoperatively compared with preoperatively. The VAS had significantly decreased at 1 day postoperatively, and the decreased sustained at the last follow-up. Therefore, PVP combined with postural reduction technique can effectively restore vertebral height, improve kyphosis, relieve pain and increase living quality of patients for the management of Kümmell’s disease. PVP with appropriate volume of the injected bone cement can effectively restore strength and stability of the fractured vertebral body.

Currently, there is no uniform standard for the amount of bone cement injection. Some scholars believed that the lower dose of bone cement can restore the mechanical properties of the fractured vertebral body, and the volume of bone cement has no obvious correlation with the analgesic effect [13]. 1.5 ml bone cement injected in the fractured vertebral body can obtain satisfactory analgesic effect [14]. However, some scholars held the opposite view that bone cement should be injected to make a higher filling rate of the vertebral body, which helps to restore the strength and rigidity of the vertebral body and obtains better clinical outcomes [15]. Besides, the volume of bone cement perfusion was related to the analgesic effect [16]. The analgesic effect of PVP was mainly due to the restoration of the stability of the fractured vertebrae after the strengthening of bone cement [17]. Furthermore, the strength of the fractured vertebral body can be restored by filling about 2 ml bone cement or 16% of the body volume, and the stiffness can be restored by filling about 4 ml bone cement or 24% of the vertebral body volume [18]. In our study, the bone cement that was injected by the side opening guide needle can be injected into the fissures and cavities of the vertebral body. The injection volume was 2.7-8 ml, with an average of 5.38 ± 1.33 ml, which had reached the requirement of restoring the strength and stiffness of the vertebral body. So, the pain relief was satisfactory after the operation.

Kümmell’s disease is a special type of osteoporotic vertebral compression fractures. The cracks and cavities will expand or compress as the body position moves. So, the postural reduction can effectively perform the reduction of fractured vertebral body and restore its height without further use of the balloon to expand the vertebral body. Moreover, the severely compressed Kümmell’s disease, after balloon dilation, will further destroy the integrity of the vertebral bone and increase the number of cracks, which may increase the risk of bone cement leakage [19]. Zhang et al. [19] compared and evaluated the safety and efficacy of PVP and percutaneous kyphoplasty (PKP) for the management of Kummell’s disease, which found that the correction of Cobb’s angle between two groups had no significant differences. Furthermore, Yu et al. [12] found that cement leakage occurred in PKP group (3/13 cases) was higher than in PVP group (1 cases/7 cases). The priming volume of cement in PVP group (6.40 ± 0.94 ml) also larger than in PKP group (5.46 ± 1.09 ml). The vertebral height restoration and kyphotic improvement, VAS of low back pain and ODI were no significant differences between two groups. Zhang et al. [20] reported that the VAS and anterior vertebral height in both PVP group and PKP group had significantly improved at 1-day postoperatively, and the improvement sustained at the final follow-up, but there were no significant differences between the PVP and PKP groups. Therefore, the postural reduction can effectively restore the degree of vertebral compression and kyphosis. PKP, compared with PVP, has no obvious advantages in restoring vertebral height, correcting kyphosis and relieving pain on the treatment of Kümmell’s disease. PVP is more economical and can be considered a preferred method of treatment with more clinical value.

The most common complication of PVP is bone cement leakage. It is easy for the bone cement to leak through the vertebral veins, fractured cracks, but causing clinical symptoms is less common [21]. The amount of bone cement injected was positively correlated with the occurrence of bone cement leakage [22]. However, some scholars believed that only the degree of compression of the vertebral body before surgery and the presence of fissures in the cortical bone were related factors of bone cement leakage [13]. In our experience, accurate puncture intraoperatively is an effective measure to prevent bone cement leakage. During the operation, it is forbidden to break through the inner wall of the pedicle into the spinal canal for the pursuit of the so-called optimal position. The prevention of the too thinning bone cement, a proper amount of bone cement, and the repeating fluoroscopy intraoperatively can effectively reduce the incidence of bone cement leakage. When the bone cement was fully dispersed in the vertebral body and continuing injection could not be further spread, surgeon should terminate the injection of bone cement.

PVP can quickly relieve pain for most patients with acute or subacute osteoporotic vertebral fractures [8]. Park et al. [23] reported that the mean VAS score significantly decreased after PVP and was maintained through to the final follow-up. However, cement leakage was observed in 26.3% without clinical symptoms. Ren et al. [7] observed that the VAS scores in both unipedicular and bipedicular PVP were lowered at 1d postoperatively, 3 months postoperatively and at the final follow-up compared to in pre-operation. In our study, the VAS scores were significantly improved at each time points postoperatively compared with in pre-operation, which confirmed that PVP surgery has a definite and sustained analgesic effect for the treatment of Kümmell’s disease.

Limitations of this study included retrospective nature, low patient numbers, single center, lack of control, and randomization. More and multi-center clinical studies are still needed to further evaluate this method and compare it to PKP control method or non-operative group.

## Conclusion

PVP has the advantages of short operation time, small trauma, quick recovery, and easy acceptance by patients. PVP surgery can effectively relieve pain and improve living quality of patients for the management of Kümmell’s disease. Combined with postural reduction technique, PVP can restore vertebra height and correct kyphosis in some degree. But its long-term efficacy is still worth observing.

## Data Availability

The datasets used and/or analysed during the current study are available from the corresponding author on reasonable request.

## Abbreviations

PKP: Percutaneous kyphoplasty
PVP: Percutaneous vertebroplasty
